# Deep Learning-Based Delayed PET Image Synthesis from Corresponding Early Scanned PET for Dosimetry Uptake Estimation

**DOI:** 10.3390/diagnostics13193045

**Published:** 2023-09-25

**Authors:** Kangsan Kim, Byung Hyun Byun, Ilhan Lim, Sang Moo Lim, Sang-Keun Woo

**Affiliations:** 1 Division of Applied RI, Korea Institute of Radiological and Medical Sciences, Seoul 01812, Republic of Korea; krmount@kirams.re.kr; 2 Department of Nuclear Medicine, Korea Institute of Radiological and Medical Sciences, Seoul 01812, Republic of Korea

**Keywords:** functional imaging, ^18^F-FDG, diagnostic radiopharmaceutical, positron emission tomography, standardized uptake value, internal dosimetry, image-to-image translation, generative adversarial network, deep learning

## Abstract

The acquisition of in vivo radiopharmaceutical distribution through imaging is time-consuming due to dosimetry, which requires the subject to be scanned at several time points post-injection. This study aimed to generate delayed positron emission tomography images from early images using a deep-learning-based image generation model to mitigate the time cost and inconvenience. Eighteen healthy participants were recruited and injected with [^18^F]Fluorodeoxyglucose. A paired image-to-image translation model, based on a generative adversarial network (GAN), was used as the generation model. The standardized uptake value (SUV) mean of the generated image of each organ was compared with that of the ground-truth. The least square GAN and perceptual loss combinations displayed the best performance. As the uptake time of the early image became closer to that of the ground-truth image, the translation performance improved. The SUV mean values of the nominated organs were estimated reasonably accurately for the muscle, heart, liver, and spleen. The results demonstrate that the image-to-image translation deep learning model is applicable for the generation of a functional image from another functional image acquired from normal subjects, including predictions of organ-wise activity for specific normal organs.

## 1. Introduction

The internal dosimetry of radiopharmaceuticals is necessary to predict their toxicity and develop treatment plans [1,2]. Their cumulative radioactivity allows them to be potential therapeutic substitutes, exhibiting the same targeting vectors as peptides or monoclonal antibodies; thus, the internal dosimetry of radiopharmaceuticals for diagnosis has been conducted in various studies. To estimate the absorbed doses, defined as the deposited energy per unit mass of the region of interest, it is necessary to acquire a spatial map of the radiopharmaceutical and the S-value, defined as the mean dose rate absorbed by the target region from the unit radioactivity at the source region [2,3]. Although some studies have aimed to calculate personalized S-values based on deep learning, the general approach is to utilize pre-calculated data through a voxel phantom model against various clinical conditions, including age and sex.

The in vivo distribution of radiopharmaceuticals can be determined through reconstructive three-dimensional (3D) quantitative imaging, such as positron emission tomography (PET) and single-photon emission computed tomography (SPECT) [4]. Radioisotopes within the body gradually decay; therefore, estimating the cumulative degree of radioactivity for each organ in the body is required. This is typically calculated by integrating a time–activity curve. From the exponential behavior of the radioactive decay of radioisotopes based on its random nature, a time–activity curve model can be composed from the mono- or linear combination of the exponential basis function to be fitted. The model is estimated via curve fitting with radioactivity in the region at several time points [5,6]. Hence, the acquisition of functional imaging is required for at least three time points to perform dosimetry. This is a burdensome task in both real applications and research, as radiopharmaceuticals require at least an hour to be distributed within the body after administration. Therefore, determining the in vivo distribution of a diagnostic radiopharmaceutical at specific time points post-injection using the generation method can mitigate the burden. Therefore, we proposed a deep learning model that generates a PET image of diagnostic radiopharmaceuticals demonstrating post-injection points later than an early scanned image.

Image-to-image translation (I2I) is a deep learning scheme in the computer vision field that aims to transfer an image presented in one domain to another with a distinctive style or characteristic. It has also been widely used in medical imaging [7,8] for reconstruction, segmentation [9,10,11], and cross-modality conversion [12,13,14,15,16]. However, most studies have focused on I2I translation between anatomical images, including those obtained through magnetic resonance imaging (MRI) and computed tomography (CT); there have been few approaches to generate functional images. Among the numerous I2I methodologies, we focused on those based on generative adversarial networks (GANs) [17], which have been used for data synthesis tasks for PET [18,19]. Based on conditional GANs (cGANs) [20], which enable GANs to be exploited in the translation problem, many studies [21,22] on paired I2I translation have proposed a model where the image similarity term is added to the loss function.

This study aimed to investigate the method of translating a PET image of healthy participants scanned at an early uptake point to a delayed point via a paired I2I model based on a cGAN. We used paired axial two-dimensional (2D) slices of the 3D PET image as the input, and a neural network based on the 2D input was used. Since the style difference between image domains is relatively small compared with cross-modality conversion, we compared various combinations of the adversarial and image similarity loss functions to avoid the gradient vanishing problem and enhance the generation performance. The performance under several conditions, including various uptake times and reconstruction methods, was also compared. Furthermore, regarding the functional I2I translation, the standardized uptake values (SUVs) of several organs in the delayed PET image were estimated and compared with the ground-truth SUVs.

## 2. Materials and Methods

This study was approved by the Institutional Review Board of KIRAMS (IRB No.: KIRAMS 2020-09-003) and was carried out in accordance with the Declarations of Helsinki. All participants provided informed consent. Eighteen healthy volunteers (ten males; aged 18–24) without a history of malignancy were injected with 10 mCi of [^18^F]Fluorodeoxyglucose (^18^F-FDG). PET data were acquired using a Biograph 6 PET/CT scanner (Siemens Healthineers, Erlangen, Germany). 

After 3.5 min, whole-body PET images were acquired at 5, 14, 31, and 52 min after the injection. The acquisition times of the projections at 5 and 14 min were 0.5 and 2 min per bed, respectively, while those at 31 and 52 min were both 3 min per bed. All methods were performed in accordance with the relevant guidelines and regulations. Among the patient data, 4668 (12 × 389) slices were used for training, 1556 (4 × 389) slices were used for validation, and 776 (2 × 389) slices were used for the test. The projections were reconstructed to 3D tomographic images by using filtered back-projection (FBP); ordered subset expectation maximization (OSEM) for 2D and 3D images; and the iterative TrueX algorithm. Each reconstructed PET image was used as a 2D axial slice, with 389 axial slices per image. The size of the slices was 128 × 128. The dataset was normalized in the range of [−1, 1] to enhance the training performance.

**Figure 1:**
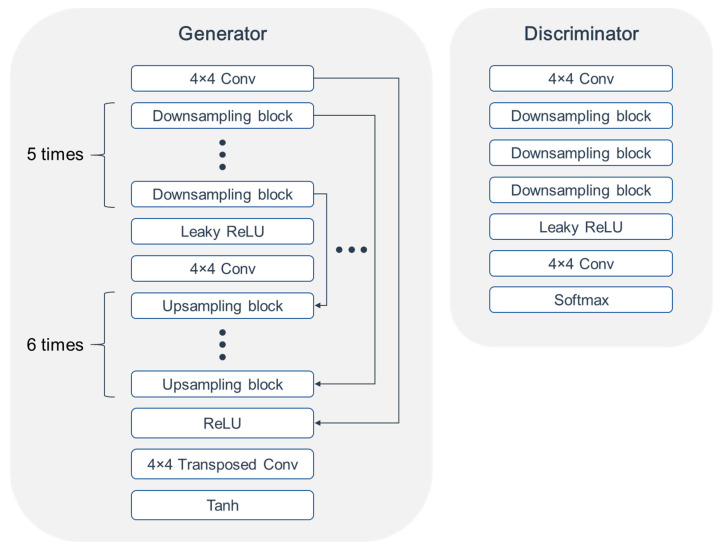
Network architectures of generator and discriminator. ConV, convolution block; ReLU, rectified linear unit.

The network architectures of the generator and discriminator are illustrated in Figure 1. The architecture of the generator was mainly based on the U-Net structure. The overall arrangement of the convolution block and hyperparameters related to the layer, such as kernel size, was based on the study by Radford et al. [23]. PatchGAN discriminator [24] was employed as an architecture. Unlike the general convolutional neural network (CNN)-based classifiers, which return the probability of whether the input is real or fake, the shape of the output has a 2D array, with elements indicating the realness of the local patches of input. This has the advantage of conserving the high-frequency information of the generated image.

The adversarial loss function of an original GAN [17] was formulated based on the binary cross-entropy (BCE) of the output of the discriminator. For the generator G, discriminator D, distribution of real data ($P_{d}$), and distribution of generated data ($P_{g}$) from the generator, the loss function is expressed as (1):

(1) $$L_{GAN}\left(D,G\right)=\underset{\times \sim P_{d}}{E}\left[log\left(D\left(x\right)\right)\right]+\underset{\tilde{x}\sim P_{g}}{E}\left[log\left(1-D\left(\tilde{x}\right)\right)\right],$$

The discriminator was updated to minimize the loss, which decreased the cross-entropy of the ideal and current discriminators. Simultaneously, the generator was taught to maximize the loss to make the generated data ‘look like’ they were sampled from the distribution of ground-truth data. Solving the minimax problem stands for minimizing the Jensen–Shannon divergence (JSD) between the distribution of the real data and that of the generated artificial data. However, the training of vanilla GAN causes a gradient vanishing problem, owing to the divergence of JSD in the early stage of training. Various studies have been proposed to tackle this instability.

Using the Wasserstein distance as a metric between the distributions was suggested by Arjovsky et al. [25] to mitigate instability. From the Kantorovich–Rubinstein duality, the loss function of Wasserstein GAN (WGAN) was expressed as the difference between the expectations of each distribution with the Lipschitz condition. In this study, WGAN-GP (gradient penalty) [26], a method of adding a GP term to the WGAN loss to satisfy the Lipschitz condition, was used. The loss function of WGAN-GP is formulated as (2):

(2) $$L_{WGAN-GP}\left(D,G\right)=\underset{\tilde{x}\sim P_{g}}{E}\left[D\left(\tilde{x}\right)\right]-\underset{x\sim P_{d}}{E}\left[D\left(x\right)\right]+\lambda_{gp}\underset{\tilde{x}\sim P_{\tilde{x}}}{E}\left[{\left({\left\|\nabla_{\tilde{x}}D\left(\tilde{x}\right)\right\|}_{2}-1\right)}^{2}\right]$$

Additionally, Mao et al. [27] argued that the gradient vanishing problem of GAN is due to fake samples far from the decision boundary of the discriminator. To penalize and pull the samples toward the decision boundary, they proposed the employment of the least square loss as an adversarial loss function (LSGAN), which has no singular point, unlike the cross-entropy-based function. The LSGAN is formulated as (3) and (4):

(3) $$L_{LSGAN}\left(D\right)=1/2\underset{x\sim P_{e}}{E}\left[{D\left(G\left(x\right)\right)}^{2}\right]+1/2\underset{x\sim P_{d}}{E}\left[{\left(D\left(x\right)-1\right)}^{2}\right]$$

(4) $$L_{LSGAN}\left(G\right)=1/2\underset{x\sim P_{e}}{E}\left[{\left(D\left(G\left(x\right)\right)-1\right)}^{2}\right]$$

During studies of paired I2I using a GAN framework, researchers made use of the adversarial loss and employed the additional loss to be summed considering the similarity of the generated image and corresponding ground-truth image [21,22]. In our study, two kinds of additional losses were considered to improve the performance of training. L1-loss was used to reflect the similarity between the generated data and its ground-truth data in the image domain. Compared to the mean squared error (L2-loss), using L1-loss is known to cause less blurring. The L1-loss is formulated as (5):

(5) $$L_{L1}\left(G\right)=\underset{x\sim P_{d}}{E}{\left\|G\left(x\right)-y\right\|}_{1}$$

Perceptual loss is an assessment metric that measures the distance between two images as perceived by humans. Estimated using the feature information of images, the perceptual loss is less affected by subtle displacement of the object placed on both images, and it is composed of two loss terms: feature content and style losses. The feature content loss was calculated using the sum of the normalized L2-norm of the features of two images extracted by convolutional blocks. The style loss was related to the style differences, such as the texture or pattern, between two images. We used the expression of style representation as a Gram matrix of the feature from the convolutional block [28]. The style loss was acquired by calculating the Frobenius norm of the difference between the style representations of the generated image and its ground-truth image. Combining the two feature losses, the perceptual loss was calculated as their weighted sum. Every weight in the loss functions was set to 1. The feature information of the data was calculated by exploiting the feature extraction layers of the pre-trained VGG19 CNN. The feature content, style, and perceptual loss is formatted as (6), (7), and (8), respectively, where $w_{i}^{c}$, $w_{i}^{s}$**,** α, and β are the hyperparameters for the weighted sum.

(6) $$L_{Content}\left(G\right)=\underset{\tilde{x}\sim P_{g}}{E}\sum_{i}w_{i}^{c}/C_{i}H_{i}W_{i}{\left\|V_{i}\left(G\left(x\right)\right)-V\left(y\right)\right\|}_{2}^{2},$$

(7) $$L_{Style}\left(G\right)=\underset{\tilde{x}\sim P_{g}}{E}\sum_{i}{w_{i}^{s}\left\|{Gr}_{V_{i}}\left(G\left(x\right)\right)-{Gr}_{V_{i}}\left(y\right)\right\|}_{F}^{2}$$

(8) $$L_{Perceptual}\left(G\right)=\alpha L_{Content}\left(G\right)+\beta L_{Content}\left(G\right)$$

With the various loss functions described above, the total loss function used to optimize the generator with a fixed discriminator is expressed as (9), where $L_{Adv}\in \left\{L_{GAN},L_{WGAN-GP},L_{LSGAN}\right\}$ is an adversarial loss function, $L_{Sim}\in \left\{L_{L1},L_{perceptual}\right\}$ is an image similarity loss function, and $\lambda_{Sim}\in \left\{L_{L1},L_{perceptual}\right\}$ is the weight of the image similarity loss function.

(9) $$L_{total}=L_{Adv}\left(G,\;D\right)+\lambda_{Sim}L_{Sim}(G)$$

The generated images were evaluated using the Fréchet inception distance (FID) [29]. FID is widely used to assess the similarity between the distributions of generated and real images. It indicates the Fréchet distance of two multivariate normal distributions of features of each set of real and generated data, encoded from the inception block of Inception V3 CNN, calculated as (10), where μ and C are the mean and covariance, respectively, and index d and g indicate the ground-truth and generated data, respectively.

(10) $$FID={\left\|\mu_{d}-\mu_{g}\right\|}_{2}^{2}+Tr\left(C_{d}+C_{g}-2{\left(C_{d}C_{g}\right)}^{1/2}\right)$$

The classic metric was also considered to evaluate the similarity between the given image and its ground-truth image. The axial slice images generated from the model were individually assessed using the peak signal-to-noise ratio (PSNR). For the mean (μ) and standard deviation (σ) of the image, PSNR is calculated as (11), where MSE is the mean squared error of the two images, and R is the maximum pixel intensity of the data type of the pixel; R represented unity since the pixel intensities were normalized to [−1, 1].

(11) $$PSNR\left(x,\;y\right)=10\cdot {log}_{10}\left(R^{2}/MSE\left(x,y\right)\right)$$

The size of the mini-batch in training was set to 4. Adam was used as an optimizer of both the generator and discriminator, with an initial learning rate of 0.0002, and the parameters were set to β_1_ = 0.5 and β_2_ = 0.999, respectively. The learning rate was set to decay by multiplying 0.98^epoch^ by the initial learning rate. The hyperparameters used in the weighted sum, namely, λ_L1_, λ_perceptual_, and λ_gp_, were set to 200, 0.1, and 10, respectively. Training was implemented in Python 3.8.6, and PyTorch 1.10.0 [30] was used for the deep learning framework.

## 3. Results

### 3.1. Image Generation

**Figure 2:**
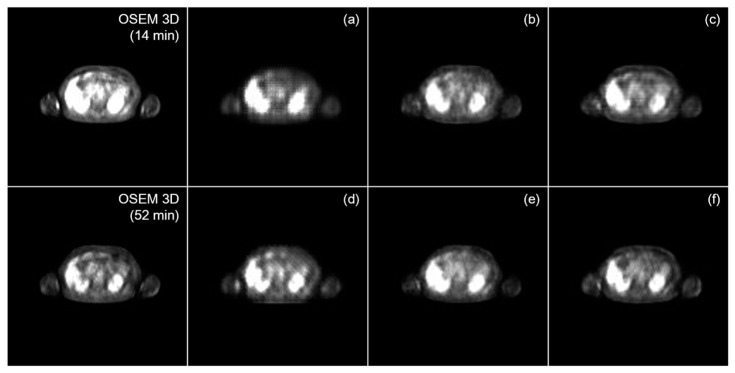
Translation results of an early PET image according to the combination of objective functions. (**a**) GAN + L1 loss; (**b**) WGAN-GP + L1 loss; (**c**) LSGAN + L1 loss; (**d**) GAN + perceptual loss; (**e**) WGAN-GP + perceptual loss; and (**f**) LSGAN + perceptual loss. 3D, three dimensional; GAN, generative adversarial network; LSGAN, least square generative adversarial network; OSEM, ordered subset expectation maximization; WGAN, Wasserstein generative adversarial network.

To compare the performance of the translation algorithm according to loss functions, we used a pair of early and delayed images obtained 14 and 52 min after the injection, reconstructed using the OSEM 3D algorithm. The generation results are plotted in Figure 2. Each plotted slice was set to have identical windows and levels. Those trained with vanilla GAN caused generated image patterns that lowered the image quality and blurred the image, regardless of the image similarity loss function, whereas the translation via WGAN-GP or LSGAN was less degraded. However, there was a slight pattern artifact within the resultant image of the combination of least square and L1-loss.

The corresponding quantitative evaluation results are listed in Table 1. For comparison, we calculated the FID between the early and delayed image distributions, which was 22.67. Vanilla GAN caused the distribution of the early image to be further from that of the ground-truth image, despite improved performance after adding perceptual loss compared to adding L1-loss. 

The loss functions using WGAN-GP and LSGAN translated the input image to be more alike to the ground-truth image. Among the combinations of adversarial and image similarity loss functions, the sum of LSGAN and perceptual loss showed the greatest performance in both FID and PSNR.

**Figure 3:**
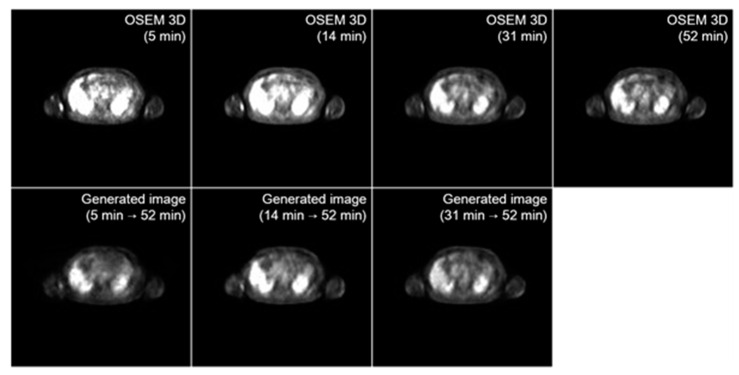
Translation results of early PET images according to the uptake time. The first row presents real images, while the images in the second row are generated from the model. Every model was trained with LSGAN + perceptual loss as its loss function. 3D, three dimensional; LSGAN, least square generative adversarial network; PET, positron emission tomography; OSEM, ordered subset expectation maximization.

The results of the I2I translation, according to the uptake time of the early image, are compared in Figure 3 and Table 1. Each image was reconstructed using OSEM 3D, and the networks were optimized with the combination of LSGAN and perceptual loss. Regarding the FID and classic metrics, the I2I translation scanned 5 min after injection, with an FID score of 21.36 and PSNR of 53.29 dB, displayed relatively worse performance, mainly for PSNR, due to the further distance between the image distribution before and after translation. However, the inception distance between the ground-truth and the generated one was also relatively insensitive regarding the input uptake time. 

**Figure 4:**
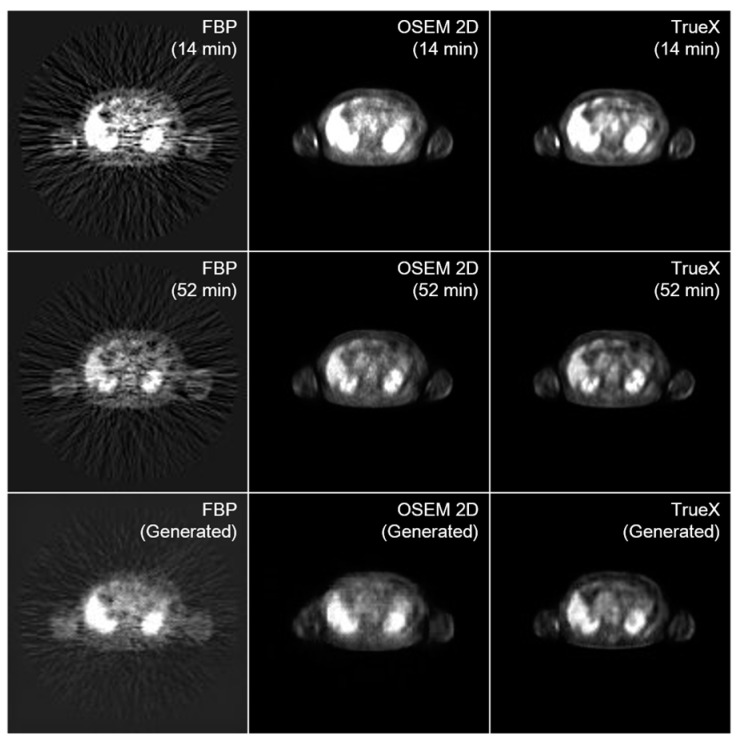
Translation results of early PET image according to the reconstruction method. Every model was trained with LSGAN + perceptual loss as its loss function. 2D, two dimensional; FBP, filtered back-projection; PET, positron emission tomography; OSEM, ordered subset expectation maximization; least square generative adversarial network.

To determine the influence of the reconstruction method, we evaluated the performance of the model trained using the images reconstructed via several methods, including FBP, OSEM 2D, OSEM 3D, and TrueX. The comparison outcomes are shown in Figure 4 and Table 1. The models trained using the images reconstructed via TrueX and OSEM 3D displayed superior outcomes. The image generated from the generation model trained with FBP images was too blurred to identify the structure due to the streak artifacts generated during reconstruction. In a quantitative sense, the results generated from the early images reconstructed via the TrueX method demonstrated the best performance in FID and PSNR, marked 10.43 and 56.87 dB, respectively. The FID and PSNR of the FBP translation were 85.93 and 40.46 dB, respectively, which were far inferior to the others.

### 3.2. SUV_mean_ Estimation

**Figure 5:**
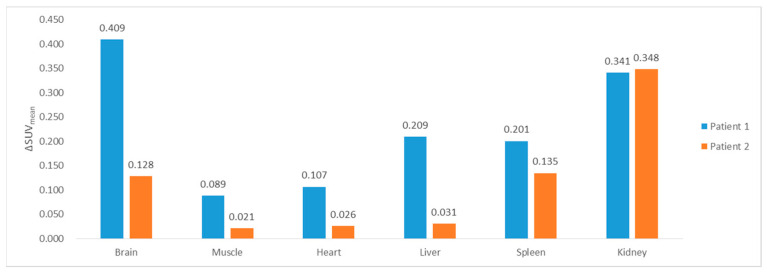
Absolute error of the SUV_mean_ of organs in generated PET images at 14 min post-injection using the I2I model, trained with the combination of LSGAN and perceptual loss function. I2I, image-to-image; LSGAN, least square generative adversarial networks; PET, positron emission tomography; SUV, standardized uptake value.

The integrated uptake values of ^18^F-FDG in the images generated 14 min post-injection were evaluated in two patients in the test dataset by comparing them with those in the delayed ground-truth PET image (Figure 5). The absolute errors of the SUV_mean_ for the muscles, heart, liver, and spleen were similar to or <0.2, while the differences in SUV_mean_ in the kidney were relatively large. The SUV_mean_ difference in the brain displayed different trends between the two patients, implying that the accuracy of the uptake prediction in the brain depended on the patient. In addition, the absolute errors of SUV_mean_ of the bladder for two patients were 9.055 and 10.63; the model could not estimate the amount of uptake in the bladder.

## 4. Discussion

We hypothesized that the selection of loss function would be significant, as the style difference between the early and delayed PET images is not clear, and GAN is widely known to suffer from unstable convergence during training. Our study found that using LSGAN or WGAN-GP mitigated the convergence issue relatively well, resulting in a finer performance than using vanilla GAN. The quantitative evaluation demonstrated that the choice of the image similarity function had less of an effect than the adversarial loss. However, the perceptual loss containing the style loss was more effective than the L1-loss, although the style difference between the two image domains was not dominant.

**Figure 6:**
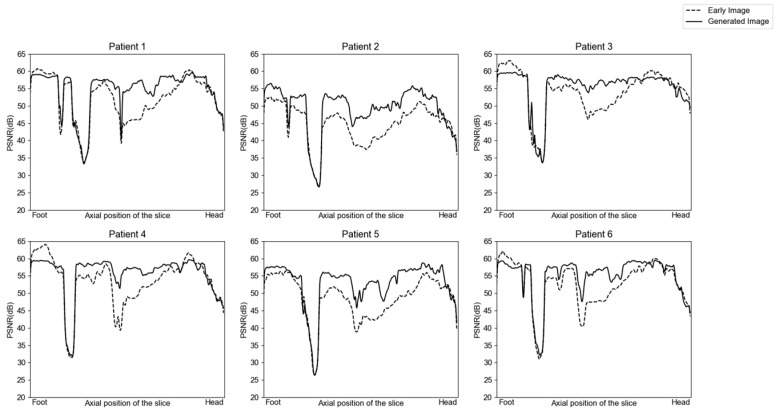
Translation performance along the axial position of the body regarding the PSNR ratio in the validation and test dataset for the 14 min post-injection as early images and 55 min post-injection as delayed image. PSNR, peak signal-to-noise ratio.

Radiopharmaceuticals injected into the body are gradually distributed via an active transport process or through the blood stream. Hence, the temporal change in activity differs along the axial slice according to the characteristics of the organs. The PSNR of the generated images was evaluated versus that of the ground-truth image acquired 52 min after injection and compared to examine the image generation performance according to the axial position. The slices between the neck and bladder could be improved regarding voxel similarity after translation (Figure 6). However, there was a little advance in the slices belonging to the head, leg, and especially the bladder, where the activity changed the most over time.

Likewise, the estimated SUV value of the generated image indicated that the training with whole-body slices resulted in the effective estimation of the SUV of organs, including the heart and liver, and a fine translation performance. However, model improvements are necessary to estimate the uptake in particular organs, such as the brain and kidney.

This study demonstrates that the deep-learning-based generation model can predict the biodistribution of a radiopharmaceutical from an early scanned image. This is clinically significant, as the high-performing deep learning model has the potential to alleviate the cost of PET or SPECT image acquisition. For instance, conventional dosimetry of radiopharmaceuticals requires multiple acquisitions for the fitting of a time–activity curve. In addition, since the distribution of a monoclonal antibody within the body is time-consuming, compared to a peptide, the application of this model is expected to be more effective for radiopharmaceutical radioimmunoconjugates (RICs). Therefore, validating the model with PET images of the diagnostic RIC is a future plan.

Moreover, even though this study examined the correspondence between the functional images of the same radiopharmaceuticals acquired at different time points post-injection, the model has the potential to be generalized for the biodistribution of two radiopharmaceuticals; it can be utilized to generate an in vivo distribution of therapeutic radiopharmaceuticals from a diagnostic one. This is significant, as the acquisition of therapeutic radiopharmaceutical distribution is hard to image, especially for an α-emitting radioisotope, including ^211^At, and ^225^Ac. Therefore, we also plan to examine the deep learning model for this purpose. Additionally, acquiring paired images of therapeutic and diagnostic radiopharmaceuticals is difficult; therefore, the unsupervised, or unpaired, I2I generation model is expected to be required.

There are some limitations to our study. First, since the model was trained with a dataset of healthy participants, it is not capable of predicting the activity of malignant tumors during delayed imaging. Therefore, additional validation carried out in patients with cancer is necessary. Second, in a methodological sense, applying the I2I model to the problem was based on the assumption that the early and delayed PET images for the same participant were ‘paired.’ However, due to the slight motion changes, including respiratory motion between two separate scans, the performance of the model may be impaired. Thus, a state-of-the-art unpaired I2I translation model or semi-supervised I2I model should be considered for application [31,32], treating the early and delayed images as unpaired, or partially paired, assuming that the slices containing the respiratory system and bladder are not paired, while the others are paired. Third, there is a downside to the 2D I2I translation model for 3D tomographic images in that the structural property along the axial direction is neglected. Therefore, we aim to improve the generation model by adopting a 3D convolution layer in the generator and discriminator. The technical improvements, expected to resolve the overparameterizations, should result in an increase in the number of parameters in the layer and a decrease in the dataset size simultaneously. Several studies have been conducted generating medical images using a 3D GAN-based generation model, dealing with the problem using patch-based schemes [32,33,34].

## 5. Conclusions

In this study, we applied a paired cGAN to PET images obtained after a relatively short uptake time of radiopharmaceuticals for I2I translation after the general uptake time. The proposed model achieved satisfactory performance using various combinations of loss functions, except for those using the cross-entropy adversarial loss. We demonstrated that a longer uptake time for the early image results in improved generation performance. Additionally, the model trained with whole-body images effectively estimated the SUV_mean_ of the heart, liver, muscle, and spleen; however, the estimated SUV_mean_ of the brain, kidney, and bladder was inaccurate. The model is expected to shorten the uptake time of radiopharmaceuticals and generate time-dependent activities by only scanning once.

## Figures and Tables

**Table 1:** Generation performance for various cases of loss functions, uptake times, and reconstruction methods.

Loss Function	Uptake Time (Min)	Reconstruction Method	FID	PSNR (dB)
GAN + L1 loss	14	OSEM 3D	45.71	52.29
WGAN + L1 loss	14	OSEM 3D	18.45	52.54
LSGAN + L1 loss	14	OSEM 3D	19.02	53.06
GAN + perceptual loss	14	OSEM 3D	31.05	51.65
WGAN + perceptual loss	14	OSEM 3D	18.69	52.86
LSGAN + perceptual loss	14	OSEM 3D	14.49	53.25
LSGAN + perceptual loss	5	OSEM 3D	21.36	53.29
LSGAN + perceptual loss	14	OSEM 3D	14.49	53.25
LSGAN + perceptual loss	31	OSEM 3D	11.61	53.75
LSGAN + perceptual loss	14	FBP	85.93	40.46
LSGAN + perceptual loss	14	OSEM 2D	15.05	52.71
LSGAN + perceptual loss	14	OSEM 3D	14.49	53.25
LSGAN + perceptual loss	14	TrueX	10.43	56.87

3D, three dimensional; GAN, generative adversarial network; FBP, FID, Fréchet inception distance; filtered back-projection; LSGAN, least square generative adversarial network; OSEM, ordered subset expectation maximization; PSNR, peak signal-to-noise ratio; WGAN, Wasserstein generative adversarial network.

## Data Availability

The data that support the findings of this study are available from the corresponding author upon reasonable request.

## References

[1] Sgouros G., Hobbs R.F. (2014). Dosimetry for Radiopharmaceutical Therapy. Semin. Nucl. Med..

[2] Graves S.A., Hobbs R.F. (2021). Dosimetry for Optimized, Personalized Radiopharmaceutical Therapy. Semin. Radiat. Oncol..

[3] Bolch W.E., Eckerman K.F., Sgouros G., Thomas S.R., Brill A.B., Fisher D.R., Howell R.W., Meredith R., Wessels B.W. (2009). MIRD Pamphlet No. 21: A Generalized Schema for Radiopharmaceutical Dosimetry—Standardization of Nomenclature. J. Nucl. Med..

[4] Hindorf C., Glatting G., Chiesa C., Lindén O., Flux G. (2010). EANM Dosimetry Committee Guidelines for Bone Marrow and Whole-Body Dosimetry. Eur. J. Nucl. Med. Mol. Imaging.

[5] Danieli R., Milano A., Gallo S., Veronese I., Lascialfari A., Indovina L., Botta F., Ferrari M., Cicchetti A., Raspanti D. (2022). Personalized Dosimetry in Targeted Radiation Therapy: A Look to Methods, Tools and Critical Aspects. J. Pers. Med..

[6] Siegel J.A., Thomas S.R., Stubbs J.B., Stabin M.G., Hays M.T., Koral K.F., Robertson J.S., Howell R.W., Wessels B.W., Fisher D.R. (1999). MIRD Pamphlet No. 16: Techniques for Quantitative Radiopharmaceutical Biodistribution Data Acquisition and Analysis for Use in Human Radiation Dose Estimates. J. Nucl. Med..

[7] Yi X., Walia E., Babyn P. (2019). Generative Adversarial Network in Medical Imaging: A Review. Med. Image Anal..

[8] Alotaibi A. (2020). Deep Generative Adversarial Networks for Image-to-Image Translation: A Review. Symmetry.

[9] Jin D., Xu Z., Tang Y., Harrison A.P., Mollura D.J. CT-Realistic Lung Nodule Simulation from 3D Conditional Generative Adversarial Networks for Robust Lung Segmentation. Proceedings of the Medical Image Computing and Computer Assisted Intervention—MICCAI 2018: 21st International Conference.

[10] Pang S., Du A., Orgun M.A., Yu Z., Wang Y., Wang Y., Liu G. (2020). CTumorGAN: A Unified Framework for Automatic Computed Tomography Tumor Segmentation. Eur. J. Nucl. Med. Mol. Imaging.

[11] Cirillo M.D., Abramian D., Eklund A. Vox2Vox: 3D-GAN for Brain Tumour Segmentation. Proceedings of the Brainlesion: Glioma, Multiple Sclerosis, Stroke and Traumatic Brain Injuries: 6th International Workshop, BrainLes 2020, Held in Conjunction with MICCAI 2020.

[12] Nie D., Trullo R., Lian J., Petitjean C., Ruan S., Wang Q., Shen D. Medical Image Synthesis with Context-Aware Generative Adversarial Networks. Proceedings of the Medical Image Computing and Computer Assisted Intervention—MICCAI 2017: 20th International Conference.

[13] Armanious K., Jiang C., Fischer M., Küstner T., Hepp T., Nikolaou K., Gatidis S., Yang B. (2020). MedGAN: Medical Image Translation Using GANs. Comput. Med. Imaging Graph..

[14] Abu-Srhan A., Almallahi I., Abushariah M.A.M., Mahafza W., Al-Kadi O.S. (2021). Paired-Unpaired Unsupervised Attention Guided GAN with Transfer Learning for Bidirectional Brain MR-CT Synthesis. Comput. Biol. Med..

[15] Cao B., Zhang H., Wang N., Gao X., Shen D. (2020). Auto-GAN: Self-Supervised Collaborative Learning for Medical Image Synthesis. Proc. AAAI Conf. Artif. Intell..

[16] Lin W., Lin W., Chen G., Zhang H., Gao Q., Huang Y., Tong T., Du M. (2021). Bidirectional Mapping of Brain MRI and PET With 3D Reversible GAN for the Diagnosis of Alzheimer’s Disease. Front. Neurosci..

[17] Goodfellow I.J., Pouget-Abadie J., Mirza M., Xu B., Warde-Farley D., Ozair S., Courville A., Bengio Y. (2014). Generative Adversarial Nets. Advances in Neural Information Processing Systems.

[18] Islam J., Zhang Y. (2020). GAN-Based Synthetic Brain PET Image Generation. Brain Inform..

[19] Abazari M.A., Soltani M., Moradi Kashkooli F., Raahemifar K. (2022). Synthetic 18F-FDG PET Image Generation Using a Combination of Biomathematical Modeling and Machine Learning. Cancers.

[20] Mirza M., Osindero S. (2014). Conditional Generative Adversarial Nets. arXiv.

[21] Isola P., Zhu J.-Y., Zhou T., Efros A.A., Research B.A. Image-To-Image Translation With Conditional Adversarial Networks. Proceedings of the IEEE Conference on Computer Vision and Pattern Recognition.

[22] Ledig C., Theis L., Huszár F., Caballero J., Cunningham A., Acosta A., Aitken A., Tejani A., Totz J., Wang Z. Photo-Realistic Single Image Super-Resolution Using a Generative Adversarial Network. Proceedings of the IEEE Conference on Computer Vision and Pattern Recognition.

[23] Radford A., Metz L., Chintala S. (2015). Unsupervised Representation Learning with Deep Convolutional Generative Adversarial Networks. arXiv.

[24] Li C., Wand M. Precomputed Real-Time Texture Synthesis with Markovian Generative Adversarial Networks. Proceedings of the Computer Vision–ECCV 2016: 14th European Conference.

[25] Arjovsky M., Chintala S., Bottou L. Wasserstein Generative Adversarial Networks. Proceedings of the International Conference on Machine Learning.

[26] Gulrajani I., Ahmed F., Arjovsky M., Dumoulin V., Courville A.C. (2017). Improved Training of Wasserstein GANs. Advances in Neural Information Processing Systems.

[27] Mao X., Li Q., Xie H., Lau R.Y.K., Wang Z., Paul Smolley S. Least Squares Generative Adversarial Networks. Proceedings of the IEEE International Conference on Computer Vision.

[28] Gatys L.A., Ecker A.S., Bethge M. (2015). A Neural Algorithm of Artistic Style. J. Vis..

[29] Heusel M., Ramsauer H., Unterthiner T., Nessler B., Hochreiter S. (2017). GANs Trained by a Two Time-Scale Update Rule Converge to a Local Nash Equilibrium. Advances in Neural Information Processing Systems.

[30] Paszke A., Gross S., Massa F., Lerer A., Bradbury Google J., Chanan G., Killeen T., Lin Z., Gimelshein N., Antiga L. (2019). PyTorch: An Imperative Style, High-Performance Deep Learning Library. Advances in Neural Information Processing Systems.

[31] Zhu J.-Y., Park T., Isola P., Efros A.A., Research B.A. Unpaired Image-To-Image Translation Using Cycle-Consistent Adversarial Networks. Proceedings of the IEEE International Conference on Computer Vision.

[32] Park T., Efros A.A., Zhang R., Zhu J.Y. Contrastive Learning for Unpaired Image-to-Image Translation. Proceedings of the Computer Vision–ECCV 2020: 16th European Conference.

[33] Lei Y., Harms J., Wang T., Liu Y., Shu H.K., Jani A.B., Curran W.J., Mao H., Liu T., Yang X. (2019). MRI-Only Based Synthetic CT Generation Using Dense Cycle Consistent Generative Adversarial Networks. Med. Phys..

[34] Klages P., Benslimane I., Riyahi S., Jiang J., Hunt M., Deasy J.O., Veeraraghavan H., Tyagi N. (2020). Patch-Based Generative Adversarial Neural Network Models for Head and Neck MR-Only Planning. Med. Phys..

